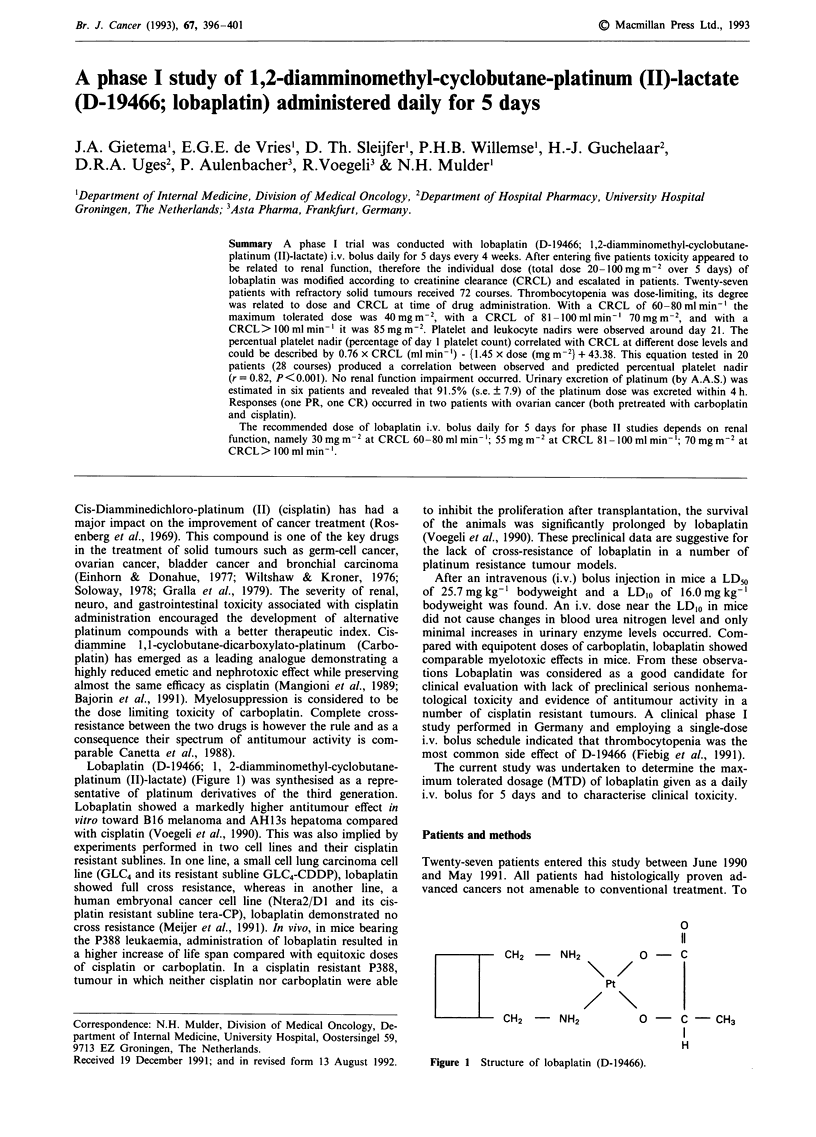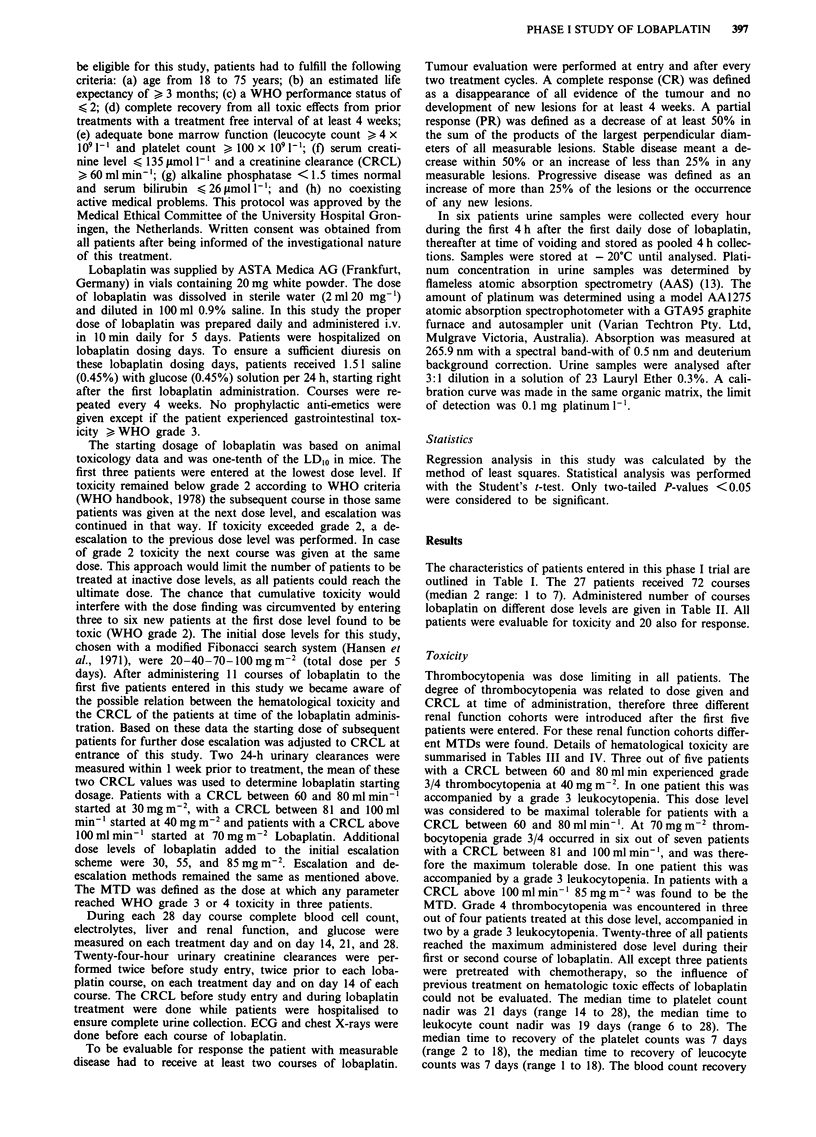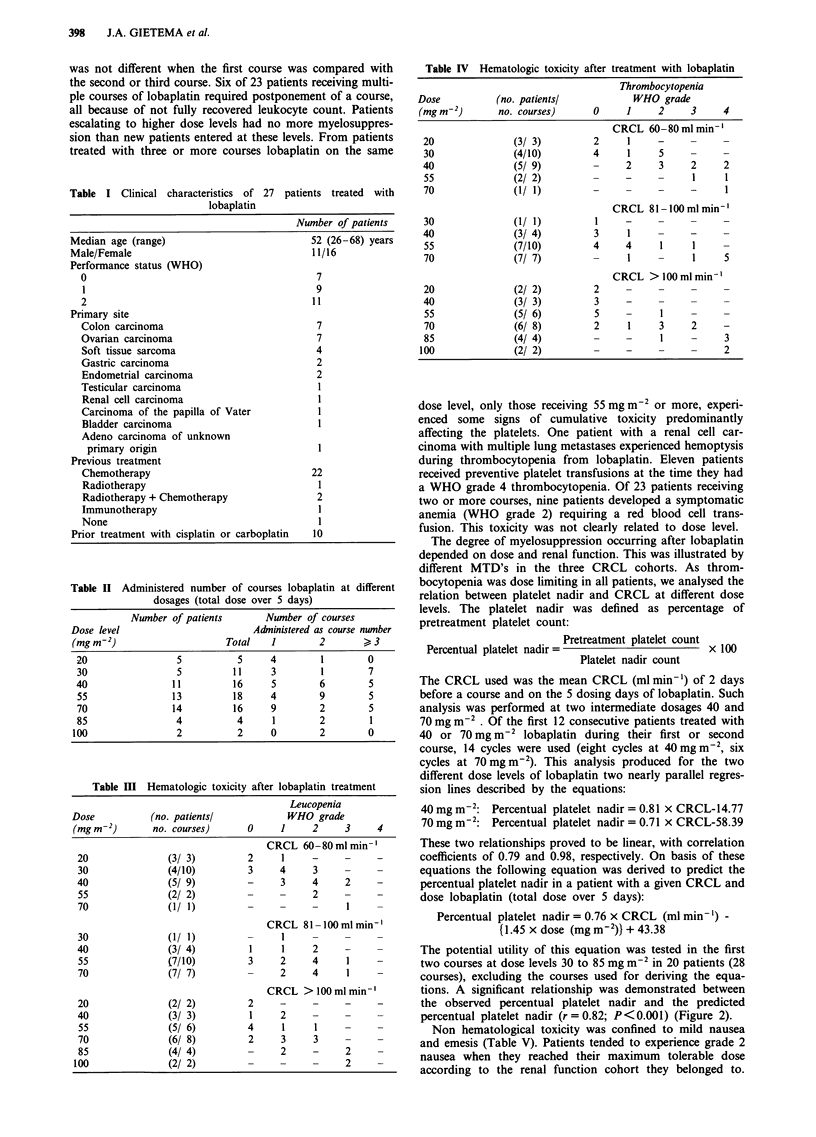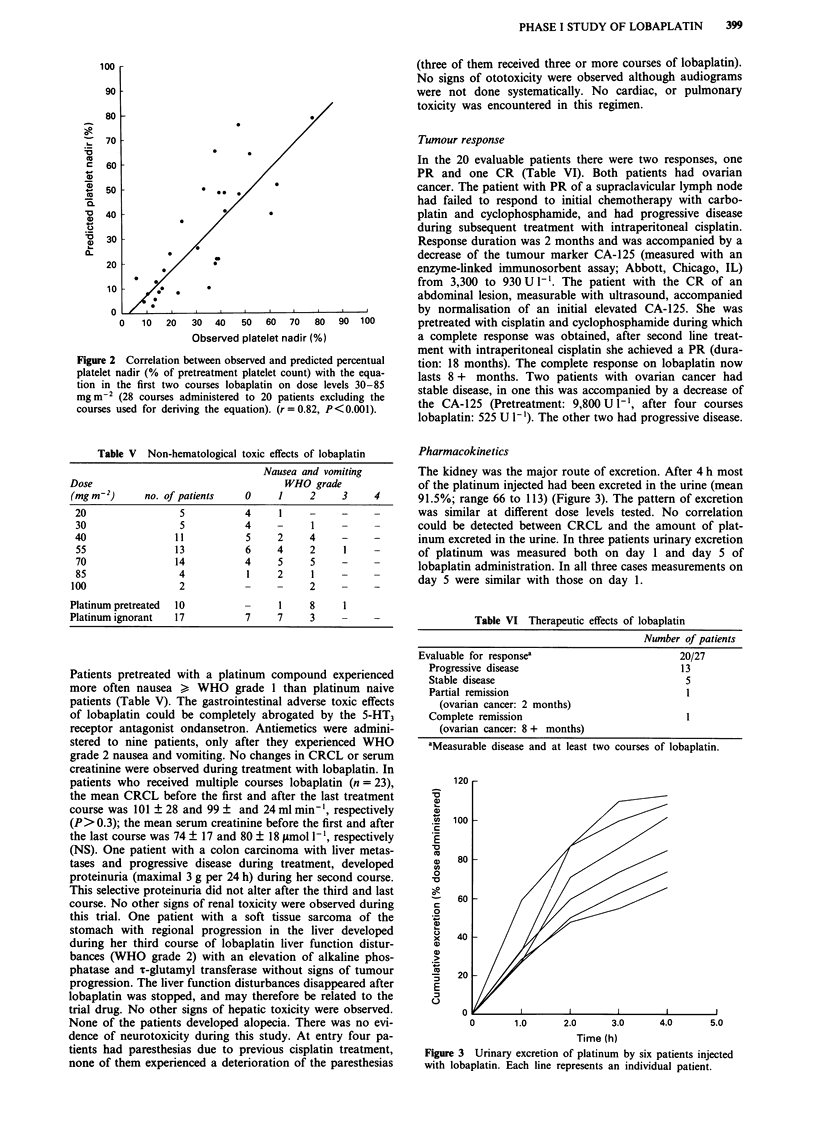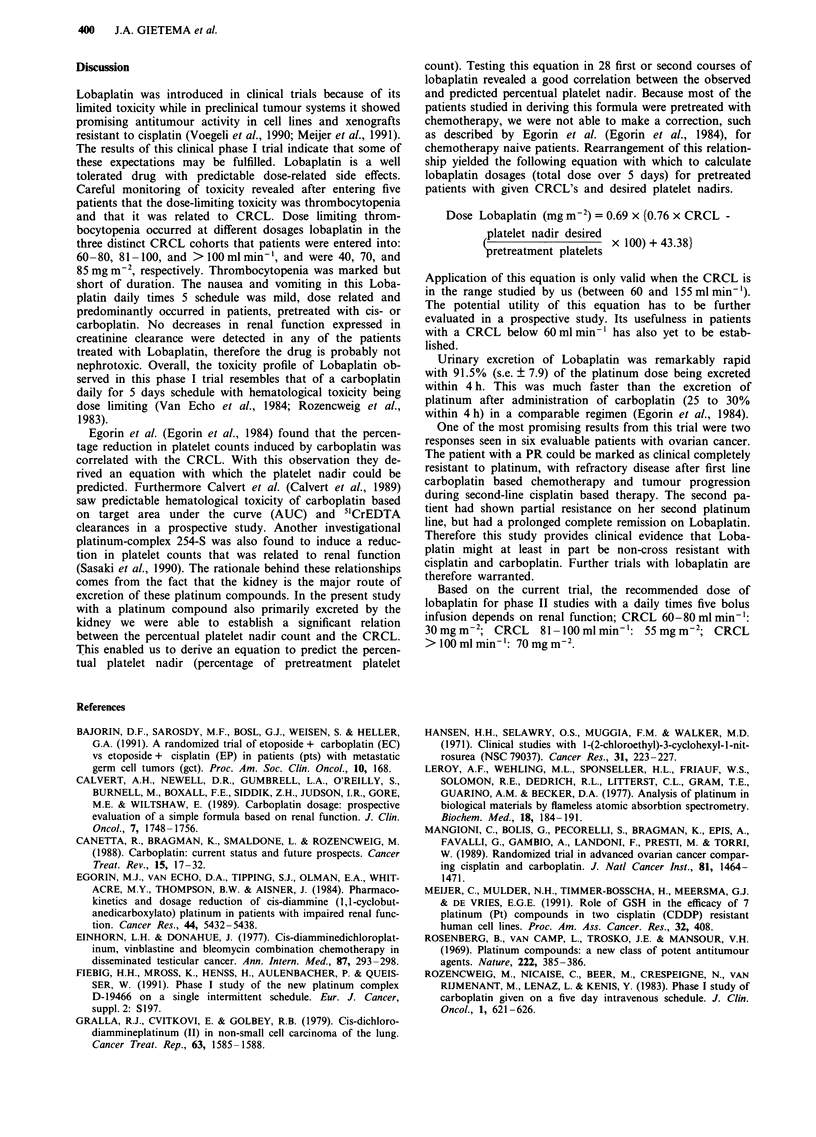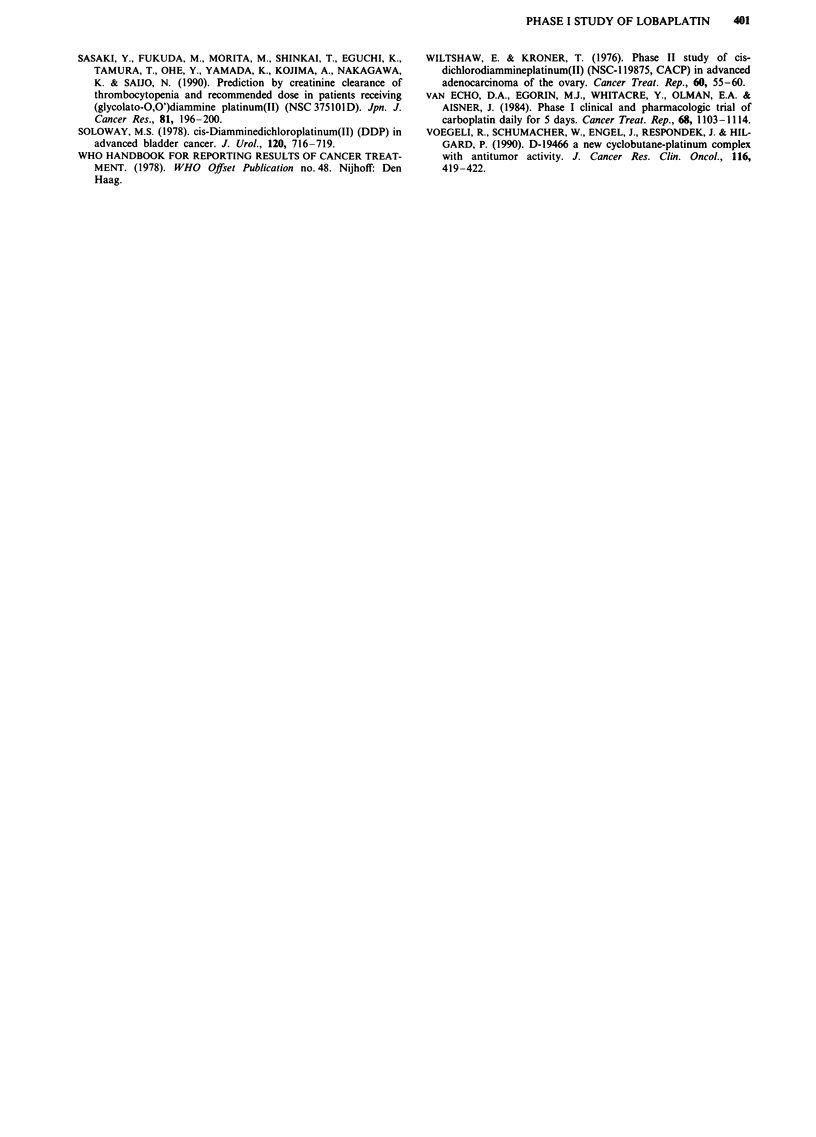# A phase I study of 1,2-diamminomethyl-cyclobutane-platinum (II)-lactate (D-19466; lobaplatin) administered daily for 5 days.

**DOI:** 10.1038/bjc.1993.73

**Published:** 1993-02

**Authors:** J. A. Gietema, E. G. de Vries, D. T. Sleijfer, P. H. Willemse, H. J. Guchelaar, D. R. Uges, P. Aulenbacher, R. Voegeli, N. H. Mulder

**Affiliations:** Department of Internal Medicine, University Hospital, Groningen, The Netherlands.

## Abstract

A phase I trial was conducted with lobaplatin (D-19466; 1,2-diamminomethyl-cyclobutane-platinum (II)-lactate) i.v. bolus daily for 5 days every 4 weeks. After entering five patients toxicity appeared to be related to renal function, therefore the individual dose (total dose 20-100 mg m-2 over 5 days) of lobaplatin was modified according to creatinine clearance (CRCL) and escalated in patients. Twenty-seven patients with refractory solid tumours received 72 courses. Thrombocytopenia was dose-limiting, its degree was related to dose and CRCL at time of drug administration. With a CRCL of 60-80 ml min-1 the maximum tolerated dose was 40 mg m-2, with a CRCL of 81-100 ml min-1 70 mg m-2, and with a CRCL > 100 ml min-1 it was 85 mg m-2. Platelet and leukocyte nadirs were observed around day 21. The percentual platelet nadir (percentage of day 1 platelet count) correlated with CRCL at different dose levels and could be described by 0.76 x CRCL (ml min-1) - (1.45 x dose (mg m-2) + 43.38. This equation tested in 20 patients (28 courses) produced a correlation between observed and predicted percentual platelet nadir (r = 0.82, P < 0.001). No renal function impairment occurred. Urinary excretion of platinum (by A.A.S) was estimated in six patients and revealed that 91.5% (s.e. +/- 7.9) of the platinum dose was excreted within 4 h. Responses (one PR, one CR) occurred in two patients with ovarian cancer (both pretreated with carboplatin and cisplatin). The recommended dose of lobaplatin i.v. bolus daily for 5 days for phase II studies depends on renal function, namely 30 mg m-2 at CRCL 60-80 ml min-1; 55 mg m-2 at CRCL 81-100 ml min-1; 70 mg m-2 at CRCL > 100 ml min-1.


### A phase I study of 1,2-diamminomethyl-cyclobutane-platinum (II)-lactate (D-19466; lobaplatin) administered daily for 5 days

J.A. Gietema[1], E.G.E. de Vries[1], D. Th. Sleijfer[1], P.H.B. Willemse[1], H.-J. Guchelaar[2], D.R.A. Uges[2], P. Aulenbacher[3], R.Voegeli[3] & N.H. Mulder[1]

[1]*Department of Internal Medicine, Division of Medical Oncology,* [2]*Department of Hospital Pharmacy, University Hospital Groningen, The Netherlands;* [3]*Asta Pharma, Frankfurt, Germany.*

**Summary** A phase I trial was conducted with lobaplatin (D-19466; 1,2-diamminomethyl-cyclobutane-platinum (II)-lactate) i.v. bolus daily for 5 days every 4 weeks. After entering five patients toxicity appeared to be related to renal function, therefore the individual dose (total dose 20–100 mg m$^{-2}$ over 5 days) of lobaplatin was modified according to creatinine clearance (CRCL) and escalated in patients. Twenty-seven patients with refractory solid tumours received 72 courses. Thrombocytopenia was dose-limiting, its degree was related to dose and CRCL at time of drug administration. With a CRCL of 60–80 ml min$^{-1}$ the maximum tolerated dose was 40 mg m$^{-2}$, with a CRCL of 81–100 ml min$^{-1}$ 70 mg m$^{-2}$, and with a CRCL > 100 ml min$^{-1}$ it was 85 mg m$^{-2}$. Platelet and leukocyte nadirs were observed around day 21. The percentual platelet nadir (percentage of day 1 platelet count) correlated with CRCL at different dose levels and could be described by 0.76 × CRCL (ml min$^{-1}$) - {1.45 × dose (mg m$^{-2}$} + 43.38. This equation tested in 20 patients (28 courses) produced a correlation between observed and predicted percentual platelet nadir ($r$ = 0.82, $P$ < 0.001). No renal function impairment occurred. Urinary excretion of platinum (by A.A.S.) was estimated in six patients and revealed that 91.5% (s.e. ± 7.9) of the platinum dose was excreted within 4 h. Responses (one PR, one CR) occurred in two patients with ovarian cancer (both pretreated with carboplatin and cisplatin).

The recommended dose of lobaplatin i.v. bolus daily for 5 days for phase II studies depends on renal function, namely 30 mg m$^{-2}$ at CRCL 60–80 ml min$^{-1}$; 55 mg m$^{-2}$ at CRCL 81–100 ml min$^{-1}$; 70 mg m$^{-2}$ at CRCL > 100 ml min$^{-1}$.

Cis-Diamminedichloro-platinum (II) (cisplatin) has had a major impact on the improvement of cancer treatment (Rosenberg *et al.*, 1969). This compound is one of the key drugs in the treatment of solid tumours such as germ-cell cancer, ovarian cancer, bladder cancer and bronchial carcinoma (Einhorn & Donahue, 1977; Wiltshaw & Kroner, 1976; Soloway, 1978; Gralla *et al.*, 1979). The severity of renal, neuro, and gastrointestinal toxicity associated with cisplatin administration encouraged the development of alternative platinum compounds with a better therapeutic index. Cis-diammine 1,1-cyclobutane-dicarboxylato-platinum (Carboplatin) has emerged as a leading analogue demonstrating a highly reduced emetic and nephrotoxic effect while preserving almost the same efficacy as cisplatin (Mangioni *et al.*, 1989; Bajorin *et al.*, 1991). Myelosuppression is considered to be the dose limiting toxicity of carboplatin. Complete cross-resistance between the two drugs is however the rule and as a consequence their spectrum of antitumour activity is comparable Canetta *et al.*, 1988).

Lobaplatin (D-19466; 1, 2-diamminomethyl-cyclobutane-platinum (II)-lactate) (Figure 1) was synthesised as a representative of platinum derivatives of the third generation. Lobaplatin showed a markedly higher antitumour effect *in vitro* toward B16 melanoma and AH13s hepatoma compared with cisplatin (Voegeli *et al.*, 1990). This was also implied by experiments performed in two cell lines and their cisplatin resistant sublines. In one line, a small cell lung carcinoma cell line ($GLC_4$ and its resistant subline $GLC_4$-CDDP), lobaplatin showed full cross resistance, whereas in another line, a human embryonal cancer cell line (Ntera2/D1 and its cisplatin resistant subline tera-CP), lobaplatin demonstrated no cross resistance (Meijer *et al.*, 1991). *In vivo*, in mice bearing the P388 leukaemia, administration of lobaplatin resulted in a higher increase of life span compared with equitoxic doses of cisplatin or carboplatin. In a cisplatin resistant P388, tumour in which neither cisplatin nor carboplatin were able to inhibit the proliferation after transplantation, the survival of the animals was significantly prolonged by lobaplatin (Voegeli *et al.*, 1990). These preclinical data are suggestive for the lack of cross-resistance of lobaplatin in a number of platinum resistance tumour models.

After an intravenous (i.v.) bolus injection in mice a $LD_{50}$ of 25.7 mg kg$^{-1}$ bodyweight and a $LD_{10}$ of 16.0 mg kg$^{-1}$ bodyweight was found. An i.v. dose near the $LD_{10}$ in mice did not cause changes in blood urea nitrogen level and only minimal increases in urinary enzyme levels occurred. Compared with equipotent doses of carboplatin, lobaplatin showed comparable myelotoxic effects in mice. From these observations Lobaplatin was considered as a good candidate for clinical evaluation with lack of preclinical serious nonhematological toxicity and evidence of antitumour activity in a number of cisplatin resistant tumours. A clinical phase I study performed in Germany and employing a single-dose i.v. bolus schedule indicated that thrombocytopenia was the most common side effect of D-19466 (Fiebig *et al.*, 1991).

The current study was undertaken to determine the maximum tolerated dosage (MTD) of lobaplatin given as a daily i.v. bolus for 5 days and to characterise clinical toxicity.

### Patients and methods

Twenty-seven patients entered this study between June 1990 and May 1991. All patients had histologically proven advanced cancers not amenable to conventional treatment. To

**Figure 1** Structure of lobaplatin (D-19466).

Correspondence: N.H. Mulder, Division of Medical Oncology, Department of Internal Medicine, University Hospital, Oostersingel 59, 9713 EZ Groningen, The Netherlands.

Received 19 December 1991; and in revised form 13 August 1992.

be eligible for this study, patients had to fulfill the following criteria: (a) age from 18 to 75 years; (b) an estimated life expectancy of $\geqslant 3$ months; (c) a WHO performance status of $\leqslant 2$; (d) complete recovery from all toxic effects from prior treatments with a treatment free interval of at least 4 weeks; (e) adequate bone marrow function (leucocyte count $\geqslant 4 \times 10^9\,l^{-1}$ and platelet count $\geqslant 100 \times 10^9\,l^{-1}$; (f) serum creatinine level $\leqslant 135\,\mu mol\,l^{-1}$ and a creatinine clearance (CRCL) $\geqslant 60\,ml\,min^{-1}$; (g) alkaline phosphatase $< 1.5$ times normal and serum bilirubin $\leqslant 26\,\mu mol\,l^{-1}$; and (h) no coexisting active medical problems. This protocol was approved by the Medical Ethical Committee of the University Hospital Groningen, the Netherlands. Written consent was obtained from all patients after being informed of the investigational nature of this treatment.

Lobaplatin was supplied by ASTA Medica AG (Frankfurt, Germany) in vials containing 20 mg white powder. The dose of lobaplatin was dissolved in sterile water ($2\,ml\,20\,mg^{-1}$) and diluted in 100 ml 0.9% saline. In this study the proper dose of lobaplatin was prepared daily and administered i.v. in 10 min daily for 5 days. Patients were hospitalized on lobaplatin dosing days. To ensure a sufficient diuresis on these lobaplatin dosing days, patients received 1.5 l saline (0.45%) with glucose (0.45%) solution per 24 h, starting right after the first lobaplatin administration. Courses were repeated every 4 weeks. No prophylactic anti-emetics were given except if the patient experienced gastrointestinal toxicity $\geqslant$ WHO grade 3.

The starting dosage of lobaplatin was based on animal toxicology data and was one-tenth of the $LD_{10}$ in mice. The first three patients were entered at the lowest dose level. If toxicity remained below grade 2 according to WHO criteria (WHO handbook, 1978) the subsequent course in those same patients was given at the next dose level, and escalation was continued in that way. If toxicity exceeded grade 2, a de-escalation to the previous dose level was performed. In case of grade 2 toxicity the next course was given at the same dose. This approach would limit the number of patients to be treated at inactive dose levels, as all patients could reach the ultimate dose. The chance that cumulative toxicity would interfere with the dose finding was circumvented by entering three to six new patients at the first dose level found to be toxic (WHO grade 2). The initial dose levels for this study, chosen with a modified Fibonacci search system (Hansen *et al.*, 1971), were 20-40-70-100 $mg\,m^{-2}$ (total dose per 5 days). After administering 11 courses of lobaplatin to the first five patients entered in this study we became aware of the possible relation between the hematological toxicity and the CRCL of the patients at time of the lobaplatin administration. Based on these data the starting dose of subsequent patients for further dose escalation was adjusted to CRCL at entrance of this study. Two 24-h urinary clearances were measured within 1 week prior to treatment, the mean of these two CRCL values was used to determine lobaplatin starting dosage. Patients with a CRCL between 60 and 80 $ml\,min^{-1}$ started at 30 $mg\,m^{-2}$, with a CRCL between 81 and 100 ml $min^{-1}$ started at 40 $mg\,m^{-2}$ and patients with a CRCL above 100 $ml\,min^{-1}$ started at 70 $mg\,m^{-2}$ Lobaplatin. Additional dose levels of lobaplatin added to the initial escalation scheme were 30, 55, and 85 $mg\,m^{-2}$. Escalation and de-escalation methods remained the same as mentioned above. The MTD was defined as the dose at which any parameter reached WHO grade 3 or 4 toxicity in three patients.

During each 28 day course complete blood cell count, electrolytes, liver and renal function, and glucose were measured on each treatment day and on day 14, 21, and 28. Twenty-four-hour urinary creatinine clearances were performed twice before study entry, twice prior to each lobaplatin course, on each treatment day and on day 14 of each course. The CRCL before study entry and during lobaplatin treatment were done while patients were hospitalised to ensure complete urine collection. ECG and chest X-rays were done before each course of lobaplatin.

To be evaluable for response the patient with measurable disease had to receive at least two courses of lobaplatin. Tumour evaluation were performed at entry and after every two treatment cycles. A complete response (CR) was defined as a disappearance of all evidence of the tumour and no development of new lesions for at least 4 weeks. A partial response (PR) was defined as a decrease of at least 50% in the sum of the products of the largest perpendicular diameters of all measurable lesions. Stable disease meant a decrease within 50% or an increase of less than 25% in any measurable lesions. Progressive disease was defined as an increase of more than 25% of the lesions or the occurrence of any new lesions.

In six patients urine samples were collected every hour during the first 4 h after the first daily dose of lobaplatin, thereafter at time of voiding and stored as pooled 4 h collections. Samples were stored at $-20°C$ until analysed. Platinum concentration in urine samples was determined by flameless atomic absorption spectrometry (AAS) (13). The amount of platinum was determined using a model AA1275 atomic absorption spectrophotometer with a GTA95 graphite furnace and autosampler unit (Varian Techtron Pty. Ltd, Mulgrave Victoria, Australia). Absorption was measured at 265.9 nm with a spectral band-with of 0.5 nm and deuterium background correction. Urine samples were analysed after 3:1 dilution in a solution of 23 Lauryl Ether 0.3%. A calibration curve was made in the same organic matrix, the limit of detection was 0.1 mg platinum $l^{-1}$.

### *Statistics*

Regression analysis in this study was calculated by the method of least squares. Statistical analysis was performed with the Student's *t*-test. Only two-tailed *P*-values $< 0.05$ were considered to be significant.

### Results

The characteristics of patients entered in this phase I trial are outlined in Table I. The 27 patients received 72 courses (median 2 range: 1 to 7). Administered number of courses lobaplatin on different dose levels are given in Table II. All patients were evaluable for toxicity and 20 also for response.

### *Toxicity*

Thrombocytopenia was dose limiting in all patients. The degree of thrombocytopenia was related to dose given and CRCL at time of administration, therefore three different renal function cohorts were introduced after the first five patients were entered. For these renal function cohorts different MTDs were found. Details of hematological toxicity are summarised in Tables III and IV. Three out of five patients with a CRCL between 60 and 80 ml min experienced grade 3/4 thrombocytopenia at 40 $mg\,m^{-2}$. In one patient this was accompanied by a grade 3 leukocytopenia. This dose level was considered to be maximal tolerable for patients with a CRCL between 60 and 80 $ml\,min^{-1}$. At 70 $mg\,m^{-2}$ thrombocytopenia grade 3/4 occurred in six out of seven patients with a CRCL between 81 and 100 $ml\,min^{-1}$, and was therefore the maximum tolerable dose. In one patient this was accompanied by a grade 3 leukocytopenia. In patients with a CRCL above 100 $ml\,min^{-1}$ 85 $mg\,m^{-2}$ was found to be the MTD. Grade 4 thrombocytopenia was encountered in three out of four patients treated at this dose level, accompanied in two by a grade 3 leukocytopenia. Twenty-three of all patients reached the maximum administered dose level during their first or second course of lobaplatin. All except three patients were pretreated with chemotherapy, so the influence of previous treatment on hematologic toxic effects of lobaplatin could not be evaluated. The median time to platelet count nadir was 21 days (range 14 to 28), the median time to leukocyte count nadir was 19 days (range 6 to 28). The median time to recovery of the platelet counts was 7 days (range 2 to 18), the median time to recovery of leucocyte counts was 7 days (range 1 to 18). The blood count recovery was not different when the first course was compared with the second or third course. Six of 23 patients receiving multiple courses of lobaplatin required postponement of a course, all because of not fully recovered leukocyte count. Patients escalating to higher dose levels had no more myelosuppression than new patients entered at these levels. From patients treated with three or more courses lobaplatin on the same dose level, only those receiving 55 mg m$^{-2}$ or more, experienced some signs of cumulative toxicity predominantly affecting the platelets. One patient with a renal cell carcinoma with multiple lung metastases experienced hemoptysis during thrombocytopenia from lobaplatin. Eleven patients received preventive platelet transfusions at the time they had a WHO grade 4 thrombocytopenia. Of 23 patients receiving two or more courses, nine patients developed a symptomatic anemia (WHO grade 2) requiring a red blood cell transfusion. This toxicity was not clearly related to dose level.

**Table I** Clinical characteristics of 27 patients treated with lobaplatin

| | *Number of patients* |
|---|---|
| Median age (range) | 52 (26–68) years |
| Male/Female | 11/16 |
| Performance status (WHO) | |
| 0 | 7 |
| 1 | 9 |
| 2 | 11 |
| Primary site | |
| Colon carcinoma | 7 |
| Ovarian carcinoma | 7 |
| Soft tissue sarcoma | 4 |
| Gastric carcinoma | 2 |
| Endometrial carcinoma | 2 |
| Testicular carcinoma | 1 |
| Renal cell carcinoma | 1 |
| Carcinoma of the papilla of Vater | 1 |
| Bladder carcinoma | 1 |
| Adeno carcinoma of unknown primary origin | 1 |
| Previous treatment | |
| Chemotherapy | 22 |
| Radiotherapy | 1 |
| Radiotherapy + Chemotherapy | 2 |
| Immunotherapy | 1 |
| None | 1 |
| Prior treatment with cisplatin or carboplatin | 10 |

**Table II** Administered number of courses lobaplatin at different dosages (total dose over 5 days)

| *Dose level (mg m$^{-2}$)* | *Number of patients* | *Number of courses Administered Total* | *as course number 1* | *2* | *≥3* |
|---|---|---|---|---|---|
| 20 | 5 | 5 | 4 | 1 | 0 |
| 30 | 5 | 11 | 3 | 1 | 7 |
| 40 | 11 | 16 | 5 | 6 | 5 |
| 55 | 13 | 18 | 4 | 9 | 5 |
| 70 | 14 | 16 | 9 | 2 | 5 |
| 85 | 4 | 4 | 1 | 2 | 1 |
| 100 | 2 | 2 | 0 | 2 | 0 |

**Table III** Hematologic toxicity after lobaplatin treatment

| *Dose (mg m$^{-2}$)* | *(no. patients/ no. courses)* | *Leucopenia WHO grade 0* | *1* | *2* | *3* | *4* |
|---|---|---|---|---|---|---|
| | | CRCL 60–80 ml min$^{-1}$ | | | | |
| 20 | (3/ 3) | 2 | 1 | – | – | – |
| 30 | (4/10) | 3 | 4 | 3 | – | – |
| 40 | (5/ 9) | – | 3 | 4 | 2 | – |
| 55 | (2/ 2) | – | – | 2 | – | – |
| 70 | (1/ 1) | – | – | – | 1 | – |
| | | CRCL 81–100 ml min$^{-1}$ | | | | |
| 30 | (1/ 1) | – | 1 | – | – | – |
| 40 | (3/ 4) | 1 | 1 | 2 | – | – |
| 55 | (7/10) | 3 | 2 | 4 | 1 | – |
| 70 | (7/ 7) | – | 2 | 4 | 1 | – |
| | | CRCL > 100 ml min$^{-1}$ | | | | |
| 20 | (2/ 2) | 2 | – | – | – | – |
| 40 | (3/ 3) | 1 | 2 | – | – | – |
| 55 | (5/ 6) | 4 | 1 | 1 | – | – |
| 70 | (6/ 8) | 2 | 3 | 3 | – | – |
| 85 | (4/ 4) | – | 2 | – | 2 | – |
| 100 | (2/ 2) | – | – | – | 2 | – |

**Table IV** Hematologic toxicity after treatment with lobaplatin

| *Dose (mg m$^{-2}$)* | *(no. patients/ no. courses)* | *Thrombocytopenia WHO grade 0* | *1* | *2* | *3* | *4* |
|---|---|---|---|---|---|---|
| | | CRCL 60–80 ml min$^{-1}$ | | | | |
| 20 | (3/ 3) | 2 | 1 | – | – | – |
| 30 | (4/10) | 4 | 1 | 5 | – | – |
| 40 | (5/ 9) | – | 2 | 3 | 2 | 2 |
| 55 | (2/ 2) | – | – | – | 1 | 1 |
| 70 | (1/ 1) | – | – | – | – | 1 |
| | | CRCL 81–100 ml min$^{-1}$ | | | | |
| 30 | (1/ 1) | 1 | – | – | – | – |
| 40 | (3/ 4) | 3 | 1 | – | – | – |
| 55 | (7/10) | 4 | 4 | 1 | 1 | – |
| 70 | (7/ 7) | – | 1 | – | 1 | 5 |
| | | CRCL > 100 ml min$^{-1}$ | | | | |
| 20 | (2/ 2) | 2 | – | – | – | – |
| 40 | (3/ 3) | 3 | – | – | – | – |
| 55 | (5/ 6) | 5 | – | 1 | – | – |
| 70 | (6/ 8) | 2 | 1 | 3 | 2 | – |
| 85 | (4/ 4) | – | – | 1 | – | 3 |
| 100 | (2/ 2) | – | – | – | – | 2 |

The degree of myelosuppression occurring after lobaplatin depended on dose and renal function. This was illustrated by different MTD's in the three CRCL cohorts. As thrombocytopenia was dose limiting in all patients, we analysed the relation between platelet nadir and CRCL at different dose levels. The platelet nadir was defined as percentage of pretreatment platelet count:

$$\text{Percentual platelet nadir} = \frac{\text{Pretreatment platelet count}}{\text{Platelet nadir count}} \times 100$$

The CRCL used was the mean CRCL (ml min$^{-1}$) of 2 days before a course and on the 5 dosing days of lobaplatin. Such analysis was performed at two intermediate dosages 40 and 70 mg m$^{-2}$ . Of the first 12 consecutive patients treated with 40 or 70 mg m$^{-2}$ lobaplatin during their first or second course, 14 cycles were used (eight cycles at 40 mg m$^{-2}$, six cycles at 70 mg m$^{-2}$). This analysis produced for the two different dose levels of lobaplatin two nearly parallel regression lines described by the equations:

40 mg m$^{-2}$: Percentual platelet nadir = 0.81 × CRCL-14.77
70 mg m$^{-2}$: Percentual platelet nadir = 0.71 × CRCL-58.39

These two relationships proved to be linear, with correlation coefficients of 0.79 and 0.98, respectively. On basis of these equations the following equation was derived to predict the percentual platelet nadir in a patient with a given CRCL and dose lobaplatin (total dose over 5 days):

$$\text{Percentual platelet nadir} = 0.76 \times \text{CRCL (ml min}^{-1}) - \{1.45 \times \text{dose (mg m}^{-2})\} + 43.38$$

The potential utility of this equation was tested in the first two courses at dose levels 30 to 85 mg m$^{-2}$ in 20 patients (28 courses), excluding the courses used for deriving the equations. A significant relationship was demonstrated between the observed percentual platelet nadir and the predicted percentual platelet nadir ($r = 0.82$; $P < 0.001$) (Figure 2).

Non hematological toxicity was confined to mild nausea and emesis (Table V). Patients tended to experience grade 2 nausea when they reached their maximum tolerable dose according to the renal function cohort they belonged to.

**Figure 2** Correlation between observed and predicted percentual platelet nadir (% of pretreatment platelet count) with the equation in the first two courses lobaplatin on dose levels 30–85 mg m$^{-2}$ (28 courses administered to 20 patients excluding the courses used for deriving the equation). ($r = 0.82$, $P < 0.001$).

**Table V** Non-hematological toxic effects of lobaplatin

| *Dose (mg m$^{-2}$)* | *no. of patients* | *Nausea and vomiting WHO grade* 0 | *1* | *2* | *3* | *4* |
|---|---|---|---|---|---|---|
| 20 | 5 | 4 | 1 | – | – | – |
| 30 | 5 | 4 | – | 1 | – | – |
| 40 | 11 | 5 | 2 | 4 | – | – |
| 55 | 13 | 6 | 4 | 2 | 1 | – |
| 70 | 14 | 4 | 5 | 5 | – | – |
| 85 | 4 | 1 | 2 | 1 | – | – |
| 100 | 2 | – | – | 2 | – | – |
| Platinum pretreated | 10 | – | 1 | 8 | 1 | |
| Platinum ignorant | 17 | 7 | 7 | 3 | – | – |

Patients pretreated with a platinum compound experienced more often nausea ⩾ WHO grade 1 than platinum naive patients (Table V). The gastrointestinal adverse toxic effects of lobaplatin could be completely abrogated by the 5-$HT_3$ receptor antagonist ondansetron. Antiemetics were administered to nine patients, only after they experienced WHO grade 2 nausea and vomiting. No changes in CRCL or serum creatinine were observed during treatment with lobaplatin. In patients who received multiple courses lobaplatin ($n = 23$), the mean CRCL before the first and after the last treatment course was 101 ± 28 and 99 ± and 24 ml min$^{-1}$, respectively ($P > 0.3$); the mean serum creatinine before the first and after the last course was 74 ± 17 and 80 ± 18 μmol l$^{-1}$, respectively (NS). One patient with a colon carcinoma with liver metastases and progressive disease during treatment, developed proteinuria (maximal 3 g per 24 h) during her second course. This selective proteinuria did not alter after the third and last course. No other signs of renal toxicity were observed during this trial. One patient with a soft tissue sarcoma of the stomach with regional progression in the liver developed during her third course of lobaplatin liver function disturbances (WHO grade 2) with an elevation of alkaline phosphatase and τ-glutamyl transferase without signs of tumour progression. The liver function disturbances disappeared after lobaplatin was stopped, and may therefore be related to the trial drug. No other signs of hepatic toxicity were observed. None of the patients developed alopecia. There was no evidence of neurotoxicity during this study. At entry four patients had paresthesias due to previous cisplatin treatment, none of them experienced a deterioration of the paresthesias (three of them received three or more courses of lobaplatin). No signs of ototoxicity were observed although audiograms were not done systematically. No cardiac, or pulmonary toxicity was encountered in this regimen.

*Tumour response*

In the 20 evaluable patients there were two responses, one PR and one CR (Table VI). Both patients had ovarian cancer. The patient with PR of a supraclavicular lymph node had failed to respond to initial chemotherapy with carboplatin and cyclophosphamide, and had progressive disease during subsequent treatment with intraperitoneal cisplatin. Response duration was 2 months and was accompanied by a decrease of the tumour marker CA-125 (measured with an enzyme-linked immunosorbent assay; Abbott, Chicago, IL) from 3,300 to 930 U l$^{-1}$. The patient with the CR of an abdominal lesion, measurable with ultrasound, accompanied by normalisation of an initial elevated CA-125. She was pretreated with cisplatin and cyclophosphamide during which a complete response was obtained, after second line treatment with intraperitoneal cisplatin she achieved a PR (duration: 18 months). The complete response on lobaplatin now lasts 8 + months. Two patients with ovarian cancer had stable disease, in one this was accompanied by a decrease of the CA-125 (Pretreatment: 9,800 U l$^{-1}$, after four courses lobaplatin: 525 U l$^{-1}$). The other two had progressive disease.

*Pharmacokinetics*

The kidney was the major route of excretion. After 4 h most of the platinum injected had been excreted in the urine (mean 91.5%; range 66 to 113) (Figure 3). The pattern of excretion was similar at different dose levels tested. No correlation could be detected between CRCL and the amount of platinum excreted in the urine. In three patients urinary excretion of platinum was measured both on day 1 and day 5 of lobaplatin administration. In all three cases measurements on day 5 were similar with those on day 1.

**Table VI** Therapeutic effects of lobaplatin

| | *Number of patients* |
|---|---|
| Evaluable for response[a] | 20/27 |
| Progressive disease | 13 |
| Stable disease | 5 |
| Partial remission (ovarian cancer: 2 months) | 1 |
| Complete remission (ovarian cancer: 8 + months) | 1 |

[a]Measurable disease and at least two courses of lobaplatin.

**Figure 3** Urinary excretion of platinum by six patients injected with lobaplatin. Each line represents an individual patient.

### Discussion

Lobaplatin was introduced in clinical trials because of its limited toxicity while in preclinical tumour systems it showed promising antitumour activity in cell lines and xenografts resistant to cisplatin (Voegeli *et al.*, 1990; Meijer *et al.*, 1991). The results of this clinical phase I trial indicate that some of these expectations may be fulfilled. Lobaplatin is a well tolerated drug with predictable dose-related side effects. Careful monitoring of toxicity revealed after entering five patients that the dose-limiting toxicity was thrombocytopenia and that it was related to CRCL. Dose limiting thrombocytopenia occurred at different dosages lobaplatin in the three distinct CRCL cohorts that patients were entered into: 60–80, 81–100, and $>100\,\mathrm{ml\,min^{-1}}$, and were 40, 70, and $85\,\mathrm{mg\,m^{-2}}$, respectively. Thrombocytopenia was marked but short of duration. The nausea and vomiting in this Lobaplatin daily times 5 schedule was mild, dose related and predominantly occurred in patients, pretreated with cis- or carboplatin. No decreases in renal function expressed in creatinine clearance were detected in any of the patients treated with Lobaplatin, therefore the drug is probably not nephrotoxic. Overall, the toxicity profile of Lobaplatin observed in this phase I trial resembles that of a carboplatin daily for 5 days schedule with hematological toxicity being dose limiting (Van Echo *et al.*, 1984; Rozencweig *et al.*, 1983).

Egorin *et al.* (Egorin *et al.*, 1984) found that the percentage reduction in platelet counts induced by carboplatin was correlated with the CRCL. With this observation they derived an equation with which the platelet nadir could be predicted. Furthermore Calvert *et al.* (Calvert *et al.*, 1989) saw predictable hematological toxicity of carboplatin based on target area under the curve (AUC) and $^{51}$CrEDTA clearances in a prospective study. Another investigational platinum-complex 254-S was also found to induce a reduction in platelet counts that was related to renal function (Sasaki *et al.*, 1990). The rationale behind these relationships comes from the fact that the kidney is the major route of excretion of these platinum compounds. In the present study with a platinum compound also primarily excreted by the kidney we were able to establish a significant relation between the percentual platelet nadir count and the CRCL. This enabled us to derive an equation to predict the percentual platelet nadir (percentage of pretreatment platelet count). Testing this equation in 28 first or second courses of lobaplatin revealed a good correlation between the observed and predicted percentual platelet nadir. Because most of the patients studied in deriving this formula were pretreated with chemotherapy, we were not able to make a correction, such as described by Egorin *et al.* (Egorin *et al.*, 1984), for chemotherapy naive patients. Rearrangement of this relationship yielded the following equation with which to calculate lobaplatin dosages (total dose over 5 days) for pretreated patients with given CRCL's and desired platelet nadirs.

$$\text{Dose Lobaplatin } (\mathrm{mg\,m^{-2}}) = 0.69 \times \{0.76 \times \text{CRCL} - (\frac{\text{platelet nadir desired}}{\text{pretreatment platelets}} \times 100) + 43.38\}$$

Application of this equation is only valid when the CRCL is in the range studied by us (between 60 and $155\,\mathrm{ml\,min^{-1}}$). The potential utility of this equation has to be further evaluated in a prospective study. Its usefulness in patients with a CRCL below $60\,\mathrm{ml\,min^{-1}}$ has also yet to be established.

Urinary excretion of Lobaplatin was remarkably rapid with 91.5% (s.e. $\pm$ 7.9) of the platinum dose being excreted within 4 h. This was much faster than the excretion of platinum after administration of carboplatin (25 to 30% within 4 h) in a comparable regimen (Egorin *et al.*, 1984).

One of the most promising results from this trial were two responses seen in six evaluable patients with ovarian cancer. The patient with a PR could be marked as clinical completely resistant to platinum, with refractory disease after first line carboplatin based chemotherapy and tumour progression during second-line cisplatin based therapy. The second patient had shown partial resistance on her second platinum line, but had a prolonged complete remission on Lobaplatin. Therefore this study provides clinical evidence that Lobaplatin might at least in part be non-cross resistant with cisplatin and carboplatin. Further trials with lobaplatin are therefore warranted.

Based on the current trial, the recommended dose of lobaplatin for phase II studies with a daily times five bolus infusion depends on renal function; CRCL 60–80 $\mathrm{ml\,min^{-1}}$: $30\,\mathrm{mg\,m^{-2}}$; CRCL 81–100 $\mathrm{ml\,min^{-1}}$: $55\,\mathrm{mg\,m^{-2}}$; CRCL $>100\,\mathrm{ml\,min^{-1}}$: $70\,\mathrm{mg\,m^{-2}}$.

### References

BAJORIN, D.F., SAROSDY, M.F., BOSL, G.J., WEISEN, S. & HELLER, G.A. (1991). A randomized trial of etoposide + carboplatin (EC) vs etoposide + cisplatin (EP) in patients (pts) with metastatic germ cell tumors (gct). *Proc. Am. Soc. Clin. Oncol.*, **10**, 168.

CALVERT, A.H., NEWELL, D.R., GUMBRELL, L.A., O'REILLY, S., BURNELL, M., BOXALL, F.E., SIDDIK, Z.H., JUDSON, I.R., GORE, M.E. & WILTSHAW, E. (1989). Carboplatin dosage: prospective evaluation of a simple formula based on renal function. *J. Clin. Oncol.*, **7**, 1748–1756.

CANETTA, R., BRAGMAN, K., SMALDONE, L. & ROZENCWEIG, M. (1988). Carboplatin: current status and future prospects. *Cancer Treat. Rev.*, **15**, 17–32.

EGORIN, M.J., VAN ECHO, D.A., TIPPING, S.J., OLMAN, E.A., WHITACRE, M.Y., THOMPSON, B.W. & AISNER, J. (1984). Pharmacokinetics and dosage reduction of cis-diammine (1,1-cyclobutanedicarboxylato) platinum in patients with impaired renal function. *Cancer Res.*, **44**, 5432–5438.

EINHORN, L.H. & DONAHUE, J. (1977). Cis-diamminedichloroplatinum, vinblastine and bleomycin combination chemotherapy in disseminated testicular cancer. *Ann. Intern. Med.*, **87**, 293–298.

FIEBIG, H.H., MROSS, K., HENSS, H., AULENBACHER, P. & QUEISSER, W. (1991). Phase I study of the new platinum complex D-19466 on a single intermittent schedule. *Eur. J. Cancer*, suppl. 2: S197.

GRALLA, R.J., CVITKOVI, E. & GOLBEY, R.B. (1979). Cis-dichlorodiammineplatinum (II) in non-small cell carcinoma of the lung. *Cancer Treat. Rep.*, **63**, 1585–1588.

HANSEN, H.H., SELAWRY, O.S., MUGGIA, F.M. & WALKER, M.D. (1971). Clinical studies with 1-(2-chloroethyl)-3-cyclohexyl-1-nitrosurea (NSC 79037). *Cancer Res.*, **31**, 223–227.

LEROY, A.F., WEHLING, M.L., SPONSELLER, H.L., FRIAUF, W.S., SOLOMON, R.E., DEDRICH, R.L., LITTERST, C.L., GRAM, T.E., GUARINO, A.M. & BECKER, D.A. (1977). Analysis of platinum in biological materials by flameless atomic absorbtion spectrometry. *Biochem. Med.*, **18**, 184–191.

MANGIONI, C., BOLIS, G., PECORELLI, S., BRAGMAN, K., EPIS, A., FAVALLI, G., GAMBIO, A., LANDONI, F., PRESTI, M. & TORRI, W. (1989). Randomized trial in advanced ovarian cancer comparing cisplatin and carboplatin. *J. Natl Cancer Inst.*, **81**, 1464–1471.

MEIJER, C., MULDER, N.H., TIMMER-BOSSCHA, H., MEERSMA, G.J. & DE VRIES, E.G.E. (1991). Role of GSH in the efficacy of 7 platinum (Pt) compounds in two cisplatin (CDDP) resistant human cell lines. *Proc. Am. Ass. Cancer. Res.*, **32**, 408.

ROSENBERG, B., VAN CAMP, L., TROSKO, J.E. & MANSOUR, V.H. (1969). Platinum compounds: a new class of potent antitumour agents. *Nature*, **222**, 385–386.

ROZENCWEIG, M., NICAISE, C., BEER, M., CRESPEIGNE, N., VAN RIJMENANT, M., LENAZ, L. & KENIS, Y. (1983). Phase I study of carboplatin given on a five day intravenous schedule. *J. Clin. Oncol.*, **1**, 621–626.

SASAKI, Y., FUKUDA, M., MORITA, M., SHINKAI, T., EGUCHI, K., TAMURA, T., OHE, Y., YAMADA, K., KOJIMA, A., NAKAGAWA, K. & SAIJO, N. (1990). Prediction by creatinine clearance of thrombocytopenia and recommended dose in patients receiving (glycolato-O,O')diammine platinum(II) (NSC 375101D). *Jpn. J. Cancer Res.*, **81**, 196–200.

SOLOWAY, M.S. (1978). cis-Diamminedichloroplatinum(II) (DDP) in advanced bladder cancer. *J. Urol.*, **120**, 716–719.

WHO HANDBOOK FOR REPORTING RESULTS OF CANCER TREATMENT. (1978). *WHO Offset Publication* no. 48. Nijhoff: Den Haag.

WILTSHAW, E. & KRONER, T. (1976). Phase II study of cis-dichlorodiammineplatinum(II) (NSC-119875, CACP) in advanced adenocarcinoma of the ovary. *Cancer Treat. Rep.*, **60**, 55–60.

VAN ECHO, D.A., EGORIN, M.J., WHITACRE, Y., OLMAN, E.A. & AISNER, J. (1984). Phase I clinical and pharmacologic trial of carboplatin daily for 5 days. *Cancer Treat. Rep.*, **68**, 1103–1114.

VOEGELI, R., SCHUMACHER, W., ENGEL, J., RESPONDEK, J. & HILGARD, P. (1990). D-19466 a new cyclobutane-platinum complex with antitumor activity. *J. Cancer Res. Clin. Oncol.*, **116**, 419–422.